# Supplementary material for: The effect of performance contingent reward prospects flexibly adapts to more versus less specific task goals
Source: Psychophysiology. 2024 Aug 29;61(12):e14678. doi: 10.1111/psyp.14678 (PMC11579227; doi:10.1111/psyp.14678)
Supplement: Supplementary file 1 — Table S1. ANOVA results for the SPN ERP component. PGG refers to p values after applying the Greenhouse–Geisser correction for sphericity assumption violations. TABLE S2. ANOVA result for mean CNV amplitudes at different frontocentral electrodes. PGG refers to p values after applying the Greenhouse–Geisser correction for sphericity assumption violations. TABLE S3. ANOVA result for the visual modality on mean CNV amplitudes at different frontocentral electrodes. PGG refers to p values after applying the Greenhouse–Geisser correction for sphericity assumption violations. TABLE S4. ANOVA result for the auditory modality on mean CNV amplitudes at different frontocentral electrodes. PGG refers to p values after applying the Greenhouse–Geisser correction for sphericity assumption violations. FIGURE S1. SPN amplitudes at different electrodes. Depicted are mean ERP amplitudes over participants and across the SPN time interval, from 500 ms prior to until the onset of the task cue, in μV. FIGURE S2. Mean CNV amplitudes at different electrodes. ERP amplitudes (in μV) are averaged over participants and across a CNV time interval, ranging from 500 ms prior to task stimulus onset until task stimulus onset. Pre., precise; impre., imprecise; bon., bonus; stan., standard. [file PSYP-61-e14678-s001.docx]

**Supplementary material**

**The effect of performance contingent reward prospects flexibly adapts to more versus less specific task goals**

**Nathalie Liegel^1^, Daniel Schneider^1^, Edmund Wascher^1^,** **Laura-Isabelle Klatt^1^ &** **Stefan Arnau^1^**

**^1^Leibniz Research Centre for Working Environment and Human Factors, Dortmund, Germany**

**1. Additional analyses: stimulus-preceding negativity (SPN)**

A repeated measures ANOVA on mean ERP amplitude in the time interval from 500 milliseconds (ms) to 1000 ms after reward cue onset (from 500 ms prior to task cue until task cue) was conducted for the electrodes F3,Fz,F4,C3,Cz and C4. The within-subject factors included were bonus (bonus vs. standard), preciseness (precisely cued vs. imprecisely cued), laterality (F3/C3 vs. Fz/Cz vs. F4/C4) and caudality (F3/Fz/F4 vs. C3/Cz/C4).

To better understand effects of laterality, the same ANOVA was repeated excluding central electrodes.

**Table 1. ANOVA results for the SPN ERP component.** P_GG_ refers to p values after applying the Greenhouse-Geisser correction for sphericity assumption violations.

|  | F3,Fz,F4,C3,Cz,C4 | | | F3,F4,C3,C4 | |
| --- | --- | --- | --- | --- | --- |
|  | F | p | p_GG_ | F | p |
| Laterality | 14.94 | <.001 | <.001 | 8.17 | .007 |
| Caudality | 59.87 | <.001 | <.001 | 57.35 | <.001 |
| Bonus | 102.23 | <.001 | <.001 | 92.90 | <.001 |
| Laterality × Caudality | 6.71 | .002 | .004 | 19.85 | <.001 |
| Laterality × Bonus | 5.54 | .006 | .007 | .14 | .71 |
| Caudality × Bonus | 26.31 | <.01 | <.01 | 29.53 | <.001 |
| Laterality × Caudality × Bonus | .26 | .77 | .73 | .19 | .67 |


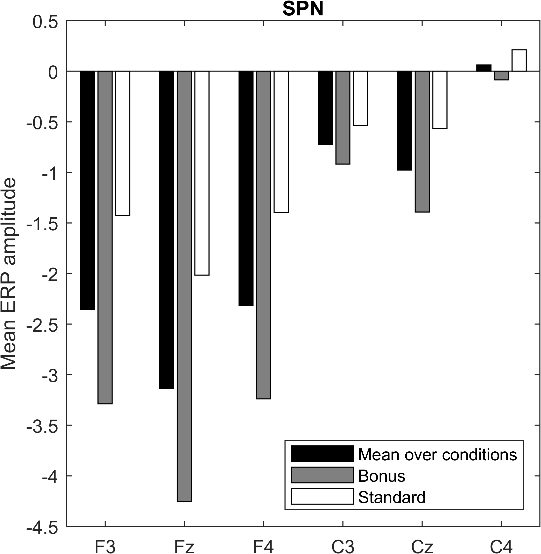


**Figure 1. SPN amplitudes at different electrodes.** Depicted are mean ERP amplitudes over participants and across the SPN time interval, from 500 ms prior to until the onset of the task cue, in µV.

**Discussion**

In line with the results reported in the main results section of the paper, ANOVA results (see table 1 and figure 1, supplementary material) indicated that SPN bonus effects were strongest at frontal electrodes, showed no hemispheric preponderance and are stronger over central than lateral electrodes.

ANOVA results further revealed a left hemispheric preponderance of SPN amplitude, primarily at central electrodes. Previous studies on instruction SPN have shown left hemispheric preponderance (Rösler, 1991, Exp. III), right hemispheric preponderance (Rösler, 1991, Exp. II), or bilateral symmetry (van Boxtel & Brunia, 1994). These findings align with the observation that the scalp distribution of the SPN prior to an instruction stimulus may depend on stimulus type (Hillman et al., 2000). Thus, our results are consistent with these earlier studies.

**2. Additional analyses: contingent negative variation (CNV)**

**2.1 Modality-unspecific analyses**

A repeated measures ANOVA on mean ERP amplitudes in the time interval from 1500 ms to 2000 ms after reward cue onset (from 500 ms prior to task stimulus onset until task stimulus) was conducted for the electrodes F3,Fz,F4,C3,Cz and C4. The within-subject factors included were bonus (bonus vs. standard), preciseness (precisely cued vs. imprecisely cued), laterality (F3/C3 vs. Fz/Cz vs. F4/C4) and caudality (F3/Fz/F4 vs. C3/Cz/C4).

To better understand effects of laterality, the same ANOVA was repeated excluding central electrodes.

**Table 2: ANOVA result for mean CNV amplitudes at different frontocentral electrodes**. P_GG_ refers to p values after applying the Greenhouse-Geisser correction for sphericity assumption violations.

|  | F3,Fz,F4,C3,Cz,C4 | | | F3,F4,C3,C4 | |
| --- | --- | --- | --- | --- | --- |
|  | F | p | p_GG_ | F | p |
| Laterality | 47.82 | <.001 | <.001 | 5.36 | .027 |
| Caudality | 24.47 | <.001 | <.001 | 28.45 | < .001 |
| Bonus | 46.37 | <.001 | <.001 | 42.14 | < .001 |
| Preciseness | 44.89 | <.001 | <.001 | 38.71 | < .001 |
| Laterality × Caudality | 1.24 | .297 | .292 | 4.54 | .041 |
| Laterality × Bonus | 5.07 | .009 | .010 | .28 | .060 |
| Caudality × Bonus | .91 | .347 | .347 | .57 | .454 |
| Laterality × Preciseness | 5.45 | .007 | .008 | 5.26 | .029 |
| Caudality × Preciseness | 10.57 | .003 | .003 | 11.99 | .002 |
| Bonus × Preciseness | 5.35 | .028 | .028 | 1.77 | .194 |
| Laterality × Caudality × Bonus | .55 | .581 | .565 | .30 | .588 |
| Laterality × Caudality × Preciseness | .40 | .675 | .657 | .64 | .430 |
| Laterality × Bonus × Preciseness | 4.50 | .015 | .016 | .11 | .743 |
| Caudality × Bonus × Preciseness | .004 | .949 | .949 | .56 | .458 |
| Laterality × Caudality × Bonus × Preciseness | 4.86 | .011 | .012 | 2.29 | .141 |

**
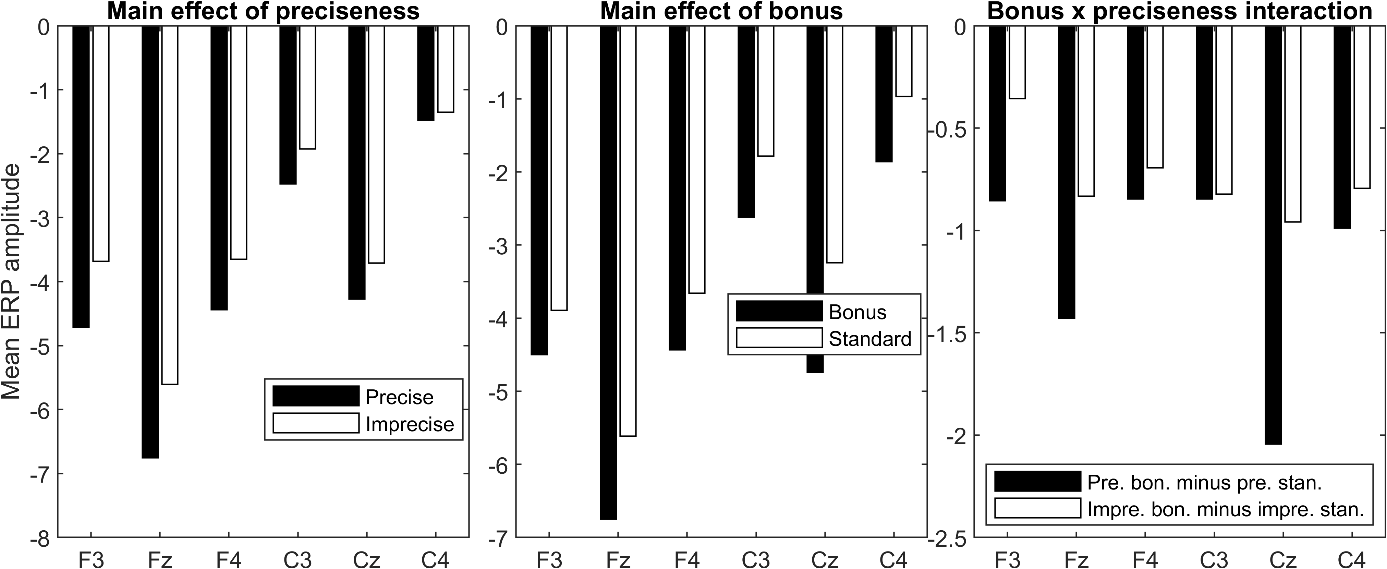
**

**Figure 2. Mean CNV amplitudes at different electrodes**. ERP amplitudes (in µV) are averaged over participants and across a CNV time interval, ranging from 500 ms prior to task stimulus onset until task stimulus onset. Pre. = precise, impre. = imprecise, bon. = bonus, stan. = standard.

**Discussion:**

The additional analyses conducted here revealed the same result pattern for bonus and preciseness effects as reported in the manuscript: Strong main effects of both, the factor Bonus and the factor Preciseness were found, as well as an interaction between the two. Bonus effects, as well as the interaction effect were stronger at central than lateral sites, both at frontal and central electrodes. In line with the results reported in the main text of the paper (see figure 7, results section), the SPN bonus effect is more frontal than the CNV bonus effect (see figure 2 and table 2, supplementary material). Preciseness effects are stronger at frontal than central electrodes and show a small but significant left hemispheric preponderance.

The CNV amplitude in our paradigm is more negative at frontal than at central electrodes, as well as more negative over the left than over the right hemisphere. This is well in line with the assumption that CNV topography depends on paradigm and stimulus type (Brunia et al., 2011; Kononowicz & Penney, 2016). It has been for example shown that slow wave topography differed for verbal, spatial or color information (Heil et al., 1997; Rösler et al., 1995).

**2.2 Modality-specific analyses**

To ensure that the reported effects were not driven by a single modality, analyses were also conducted separately for auditory and visual modalities: Two repeated measures ANOVA on mean ERP amplitude in the time interval from 500 ms to 1000 ms after reward cue onset (from 500 ms prior to until task cue onset) were conducted for the electrodes F3,Fz,F4,C3,Cz and C4. The within-subject factors included were bonus (bonus vs. standard), preciseness (precisely cued - either auditory or visual vs. imprecisely cued), laterality (F3/C3 vs. Fz/Cz vs. F4/C4) and caudality (F3/Fz/F4 vs. C3/Cz/C4). To better understand effects of laterality, the same ANOVA was repeated excluding central electrodes, respectively.

**Table 3: ANOVA result for the visual modality** on mean CNV amplitudes at different frontocentral electrodes. P_GG_ refers to p values after applying the Greenhouse-Geisser correction for sphericity assumption violations.

|  | F3,Fz,F4,C3,Cz,C4 | | | F3,F4,C3,C4 | |
| --- | --- | --- | --- | --- | --- |
|  | F | p | p_GG_ | F | p |
| Laterality | 45.92 | <.001 | <.001 | 3.31 | .079 |
| Caudality | 23.88 | <.001 | <.001 | 28.34 | <.001 |
| Bonus | 49.85 | <.001 | <.001 | 45.89 | <.001 |
| Preciseness | 18.25 | <.001 | <.001 | 13.77 | <.001 |
| Laterality × Caudality | 1.01 | .369 | .357 | 3.19 | .084 |
| Laterality × Bonus | 4.06 | .022 | .022 | .47 | .496 |
| Caudality × Bonus | .63 | .432 | .432 | .23 | .632 |
| Laterality × Preciseness | 2.04 | .139 | .140 | .99 | .328 |
| Caudality × Preciseness | 5.31 | .028 | .028 | 6.65 | .015 |
| Bonus × Preciseness | 5.28 | .028 | .028 | 1.70 | .202 |
| Laterality × Caudality × Bonus | 1.15 | .322 | .317 | 1.33 | .258 |
| Laterality × Caudality × Preciseness | .17 | .840 | .807 | .01 | .932 |
| Laterality × Bonus × Preciseness | 3.21 | .047 | .051 | < .01 | .962 |
| Caudality × Bonus × Preciseness | .01 | .912 | .912 | .94 | .340 |
| Laterality × Caudality × Bonus × Preciseness | 4.34 | .017 | .018 | .65 | .426 |

**Table 4: ANOVA result for the auditory modality** on mean CNV amplitudes at different frontocentral electrodes. P_GG_ refers to p values after applying the Greenhouse-Geisser correction for sphericity assumption violations.

|  | F3,Fz,F4,C3,Cz,C4 | | | F3,F4,C3,C4 | |
| --- | --- | --- | --- | --- | --- |
|  | F | p | p_GG_ | F | p |
| Laterality | 46.90 | <.001 | <.001 | 7.40 | .011 |
| Caudality | 24.47 | <.001 | <.001 | 27.70 | <.001 |
| Bonus | 37.99 | <.001 | <.001 | 31.19 | <.001 |
| Preciseness | 38.76 | <.001 | <.001 | 39.91 | <.001 |
| Laterality × Caudality | 1.50 | .231 | .234 | 5.76 | .023 |
| Laterality × Bonus | 5.05 | .009 | .012 | .10 | .749 |
| Caudality × Bonus | .92 | .344 | .344 | .86 | .360 |
| Laterality × Preciseness | 6.05 | .004 | .005 | 7.97 | .008 |
| Caudality × Preciseness | 10.91 | .002 | .002 | 10.08 | .003 |
| Bonus × Preciseness | 4.02 | .054 | .054 | 1.16 | .289 |
| Laterality × Caudality × Bonus | .16 | .856 | .842 | < .01 | .931 |
| Laterality × Caudality × Preciseness | 1.58 | .215 | .216 | 2.26 | .143 |
| Laterality × Bonus × Preciseness | 3.63 | .032 | .036 | .26 | .613 |
| Caudality × Bonus × Preciseness | .03 | .859 | .859 | .13 | .723 |
| Laterality × Caudality × Bonus × Preciseness | 3.23 | .046 | .047 | 3.35 | .077 |

**Discussion**

As in the modality-unspecific analyses, the result pattern observed in the cluster-based permutation test remains consistent when analyzing auditory and visual modalities separately (see table 3 and 4, supplementary material): Strong main effects of bonus and preciseness were obtained. Additionally, an interaction between both factors was found to be significant at central, but not at more lateral electrodes. The lack of significant laterality x bonus interactions when central electrodes were excluded (table 3 and 4, right panels) indicates that modality-specific bonus effects do not significantly differ between hemispheres. This is in line with the modality-unspecific results obtained above and the results of the cluster-based permutation test reported in the results section.

**References**

Brunia, C. H. M., van Boxtel, G. J. M., & Böcker, K. B. E. (2011). Negative Slow Waves as Indices of Anticipation: The Bereitschaftspotential, the Contingent Negative Variation, and the Stimulus-Preceding Negativity. In E. S. Kappenman & S. J. Luck (Hrsg.), *The Oxford Handbook of Event-Related Potential Components* (S. 0). Oxford University Press. https://doi.org/10.1093/oxfordhb/9780195374148.013.0108

Heil, M., Rösler, F., & Hennighausen, E. (1997). Topography of brain electrical activity dissociates the retrieval of spatial versus verbal information from episodic long-term memory in humans. *Neuroscience Letters*, *222*(1), 45–48. https://doi.org/10.1016/S0304-3940(97)13338-9

Hillman, C. H., Apparies, R. J., & Hatfield, B. D. (2000). Motor and nonmotor event-related potentials during a complex processing task. *Psychophysiology*, *37*(6), 731–736.

Kononowicz, T. W., & Penney, T. B. (2016). The contingent negative variation (CNV): Timing isn’t everything. *Current Opinion in Behavioral Sciences*, *8*, 231–237. https://doi.org/10.1016/j.cobeha.2016.02.022

Rösler, F. (1991). Perception or action: Some comments on preparatory negative potentials. *Electroencephalography and Clinical Neurophysiology. Supplement*, *42*, 116–129.

Rösler, F., Heil, M., & Hennighausen, E. (1995). Distinct Cortical Activation Patterns during Long-Term Memory Retrieval of Verbal, Spatial, and Color Information. *Journal of Cognitive Neuroscience*, *7*(1), 51–65. https://doi.org/10.1162/jocn.1995.7.1.51

van Boxtel, G. J., & Brunia, C. H. (1994). Motor and non-motor aspects of slow brain potentials. *Biological Psychology*, *38*(1), 37–51. https://doi.org/10.1016/0301-0511(94)90048-5
